# Does the Covid-19 pandemic affect ankle fracture incidence? Moderate decrease in Sweden

**DOI:** 10.1080/17453674.2021.1907517

**Published:** 2021-04-06

**Authors:** Emilia Möller Rydberg, Michael Möller, Jan Ekelund, Olof Wolf, David Wennergren

**Affiliations:** a Institute of Clinical Sciences, Sahlgrenska Academy, University of Gothenburg , Gothenburg ;; b Department of Orthopaedics, Sahlgrenska University Hospital, Gothenburg/Mölndal;; c Centre of Registers Västra Götaland ;; d Section of Orthopaedics, Department of Surgical Sciences, Uppsala University , Uppsala ;; e Department of Orthopaedics, Uppsala University Hospital, Uppsala, Sweden

## Abstract

Background and purpose — While many other countries implemented strict regulations and restrictions for their citizens during the 1st wave of the Covid-19 pandemic, Sweden maintained a more restrained approach. The Swedish Public Health Agency emphasized individual responsibility and pushed for behavioral changes. With strict lockdown a 77% decrease in ankle fracture incidence has been reported. We investigated whether there was a change in the incidence of ankle fractures seen at 7 selected hospitals during the Covid-19 pandemic 2020.

Patients and methods — Data on all ankle fractures treated at 7 selected departments during March 15 through June 15, 2020, and for the same period in the preceding 3 years (2017–2019), was retrieved from the Swedish Fracture Register. The number of fractures during the whole period and subsequent 30-day periods were compared between 2020 and 2017–2019, including subgroup analyses of age and sex.

Results — The monthly rate of ankle fractures was reduced by 14% in 2020 (139 fractures) compared with 2017–2019 (161 fractures). Women had a 16% decrease and patients aged > 70 years had a 29% decrease. During the 1st 30-day period, a 26% decrease in fractures was seen.

Interpretation — During the 1st wave of the Covid-19 pandemic, a moderate decline in the number of ankle fractures was seen. Women and patients aged > 70 years displayed the greatest reduction. The greatest reduction in incidence of fractures was seen during the 1st 30-day period. This indicates greater adherence to government recommendations regarding social distancing in these subgroups and during the 1st month of the pandemic. Changes in ankle fracture incidence may be a measure of lockdown extent.

While many other countries implemented strict regulations and restrictions for their citizens during the 1st wave of the Covid-19 pandemic, imposing quarantine and entry bans and closing restaurants, schools, and preschools to prevent the spread, Sweden maintained a more restrained approach. The Public Health Agency of Sweden (SPHA) emphasized individual responsibility and pushed for behavioral changes instead of imposing regulations, with the aim of reducing the pace of viral transmission (Public Health Agency of Sweden 2020). From mid-March, 2020, Swedes were advised to stay at home if they had symptoms, to keep their distance and take personal responsibility (Government Offices of Sweden 2020). The only ban imposed in Sweden during the 1st months of the pandemic during the spring of 2020 was a ban on public gatherings of 50 or more people on March 27.

Ankle fractures are common in all age groups and both sexes and most often sustained after a simple same-level fall (Bergh et al. 2020, Rydberg et al. 2020). Following the recommendations imposed by the SPHA, people worked more from home, thereby eliminating travelling to work. Sports activities and competitions were cancelled on a widespread scale. A reduction in the number of ankle fractures could be expected.

Haskel et al. (2020) reported a 77% reduction in ankle fractures in New York and Lubbe et al. (2020) reported a reduction in the number of orthopedic trauma and foot injuries in Las Vegas during a 45-day period in March–April. Kuitunen et al. (2020) described a substantial decrease in Finland in the number of visits to A&E due to orthopedic conditions during the 1st wave of the pandemic.

The Swedish Fracture Register (SFR) is a national quality register that has been collecting information on fractures of all types for almost 10 years (Wennergren et al. 2015). The SFR collects data on patient characteristics, injury mechanism, fracture type, and subsequent treatment methods for fractures of all types, treated surgically as well as non-surgically. As data are entered in the register by the treating physician at the time of the injury, they can be extracted from the register without delay.

We investigated whether there was a change in the incidence of ankle fractures seen in some selected hospitals during the Covid-19 pandemic compared with the same time period during the previous 3 years. We also investigated whether a reduction in the incidence of ankle fractures was seen in sub-groups defined by age and sex, or during certain 30-day periods.

## Patients and methods

The study is based on data from the Swedish Fracture Register (SFR). Data is entered in the SFR by the responsible physician, usually at the time of presentation at the accident and emergency department. The register has almost 100% coverage, i.e., almost all departments in Sweden treating fractures are affiliated with the SFR. The completeness of fracture registration in the SFR, i.e., the number of fractures treated in each department that is registered in the SFR, is evaluated annually and several studies have evaluated the validity of fracture classification in the SFR (Juto et al. 2016, Wennergren et al. 2017, Swedish Fracture Register 2020).

This is an observational study based on data on ankle fractures, in patients aged 16 years and above, treated at a sample of orthopedic departments with a history of high completeness in their registrations in the SFR. The orthopedic departments at the hospitals in Varberg, Uddevalla/Trollhättan, Göteborg, Borås, Falun, Gävle, and Östersund have all had a completeness in their registrations of 70% or more in the past 3 years (2016–2018) and were therefore included in the study (Swedish Fracture Register 2018, 2019). The same departments also have a history of rapid fracture entry in the register (registering a substantial number of fractures within 30 days of the injury) (Swedish Fracture Register 2020).

Data was extracted from the SFR on all ankle fractures treated at the 7 departments listed above during March 15 through June 15, 2020, and the same period in the preceding 3 years (2017–2019).

### Statistics

In order to minimize the risk of overinterpreting differences in the numbers of fractures for individual years, the observed time period for 2020 was compared with the mean for the corresponding time periods in 2017–2019. Comparisons were made for the total number of fractures during the observed time period as a whole and for the 3 x 30–day periods (March 15–April 14, April 15– May 14, and May 15–June 15) respectively. Subgroup analyses included sex and age groups. Descriptive statistics are presented as means (SD), medians (range), and proportions.

Incidence rates were compared, assuming the population size was similar during the time period 2017–2020 and that the number of fractures has a Poisson distribution.

95% confidence intervals (CI) for differences in fracture incidence were obtained by approximating the Poisson distribution with the normal distribution.

All statistics were calculated with IBM SPSS 25 (IBM Corp, Armonk, NY, USA) or SAS v 9.4 (SAS Institute, Cary, NC, USA).

### Ethics, funding, and potential conflicts of interest

The study was approved by the Swedish Ethical Review Authority (August 12, 2020; reference number 2020-02783). No funding was obtained and no conflicts of interest were declared.

## Results

### Number of fractures

From March 15 to June 15, 2020, 417 patients with ankle fractures were registered. The mean age was 52 years (SD 20) and 58% were women. During the same time period in 2017–2019, 1,446 patients with ankle fractures presented at the same departments. The mean age was 54 years (SD 20) and 60% were women (Table 1).

**Table 1.** Demographic data on ankle fractures 2017–2019 and 2020

Factor	2017–2019	2020
Age, mean (SD)	54 (20)	52 (20)
median (range)	56 (16–98)	53 (16–100)
Female sex (%)	60	59

The monthly rate of ankle fractures was 139 for the observed months in 2020 and 161 in 2017–2019. This is a statistically significant decrease of 22 (CI –37 to –6) fractures/month, corresponding to a reduction of 14% for the observation period in 2020 compared with the same period in 2017–2019 (Table 2).

**Table 2.** Number of ankle fractures observed March 15 to June 15, 2017–2019 and 2020

Factor			Monthly rates			
	Observed number		2017– 2019	2020	Estimated difference (95% CI)	Change (%) from 2017–2019
	2017– 2019	2020				
Total	1,446	417	161	139	–22 (–37 to -6)	–14
Sex						
Male	574	173	64	58	–6 (–16 to 4)	–10
Female	872	244	97	81	–16 (–28 to –4)	–16
Age group						
	251	68	28	23	–5 (–12 to 1)	–19
30–49	302	108	34	36	2 (–5 to 10)	7
50–69	524	153	58	51	–7 (–17 to 2)	–12
≥ 70	369	88	41	29	–12 (–19 to –4)	–29

### Age and sex distribution

There was a statistically significant reduction in the observed number of fractures in women of 16 (CI -28 to -4) fractures/month, corresponding to a reduction of 16%. In the age group 70 years or older, the number of fractures was reduced by 12 (CI -19 to -4) fractures/month, constituting a 29% reduction (Table 2).

### Time periods

When analyzed month by month, there were 45 (CI –72 to –19) fewer ankle fractures/month during the 1st 30-day period, corresponding to a reduction of 26%. For the following 2 x 30-day periods, no statistically significant changes were observed (Table 3).

**Table 3.** Observed number of ankle fractures 2017–2019 and 2020, analyzed month by month

Period	Observed number 2017– 2019	Monthly rates			
		2017– 2019	2020	Estimated difference (95% CI)	Change (%) from 2017–2019
March 15–April 14	523	174	129	–45 (–72 to –19)	–26
April 15–May 15	431	144	144	0.3 (–27 to 28)	0.2
May 16–June 15	492	164	144	–20 (–48 to 8)	–12

A further monthly subgroup analysis grouped by sex showed a decline of 34 (CI –54 to –13) fractures/month, representing a 31% reduction in the number of fractures among women during the 1st 30-day period studied in 2020. There was no reduction for the other periods or for men at any time point (Table 4). In the age group 30–49 years an increase of 13 (CI –1 to 27) fractures/month was seen for the second 30-day period between April 15 and May 15, corresponding to an increase of 43% (Table 4). The subgroup analysis also revealed 18 (CI –31 to –4) fewer fractures/month during the 1st 30-day period in the age group of 70 years or older, constituting a reduction of 36%.

**Table 4.** Observed number of ankle fractures in 2017–2019 and 2020, analyzed month by month and sub-grouped for sex and into age groups

Period	Observed number 2017– 2019	Monthly rates			
		2017– 2019	2020	Estimated difference (95% CI)	Change (%) from 2017–2019
March 15–April 14					
Male	200	67	55	–12 (–29 to 6)	–18
Female	323	108	74	–34 (–54 to –13)	–31
April 15–May 15					
Male	160	53	56	3 (–14 to 20)	5
Female	271	90	88	–2 (–24 to 19)	–3
May 16–June 15					
Male	214	71	62	–9 (–28 to 9)	–13
Female	278	93	82	–11 (–32 to 10)	–12
March 15–April 14					
Age < 30	82	27	22	–5 (–16 to 6)	–19
Age 30–49	105	35	28	–7 (–19 to 5)	–20
Age 50–69	190	63	48	–15 (–32 to 1)	–24
Age ≥ 70	146	49	31	–18 (–31 to –4)	–36
April 15–May 15					
Age < 30	74	25	19	–6 (–16 to 5)	–23
Age 30–49	90	30	43	13 (–1 to 27)	43
Age 50–69	153	51	52	1 (–15 to 17)	2
Age ≥ 70	114	38	30	–8 (–21 to 5)	–21
May 16–June 15					
Age < 30	95	32	27	–5 (–17 to 7)	–15
Age 30–49	107	36	37	1 (–12 to 15)	4
Age 50–69	181	60	53	–7 (–24 to 9)	–12
Age ≥ 70	109	36	27	–9 (–22 to 3)	–26

### Distribution between AO fracture groups and treatment type

During the observed months 2017–2019 compared with the same time period in 2020, the distribution of fractures by AO fracture group was similar. Likewise, regarding treatment type, similar distribution was observed between surgical and non-surgical treatment (data not shown).

### Discussion

Our most important finding is a reduction in the number of ankle fractures during the 1st wave of the Covid-19 pandemic in 7 investigated departments in Sweden. The reduction occurred in women, and was most pronounced in patients aged 70 years or older, and for the 1st 30-day period of the pandemic. Women and people over the age of 70 apparently had the highest adherence.

Our findings are in line, but with a lower incidence reduction, with the findings in the recent study by Haskel et al. (2020), who reported a 77% reduction in the number of ankle fractures during the 1st month of the pandemic in a level 1 trauma center in New York City. The New York lockdown was strict: all non-essential businesses closed, discouragement of public transportation, individuals to stay at least 6 feet from one another, and social gatherings of any size prohibited, i.e. regulations much stronger than those applied in Sweden. Our study has a longer observation time and reports a reduction in the number of ankle fractures for the entire time period, as well as for the 1st month. This could be interpreted as meaning that the adherence to recommendations on social distancing was higher during the 1st month of the pandemic than during the following months. Furthermore, we found a reduction in ankle fractures in patients aged > 70 years for the entire study period, as well as the 1st 30-day period. During the 1st wave of the pandemic, the Swedish authorities issued specific recommendations for people in this age group. The observed decrease in the number of fractures could be interpreted as strict adherence to these recommendations by the elderly, especially during the period from mid-March to mid-April. This is supported by the findings by Ponkilainen et al. (2020) reporting a decrease in the number of hip fracture patients, interpreted as a good adherence from senior citizens to recommendations on social distancing and lessening of activities in Finland.

In Hong Kong, there was a 20% decrease in surgically treated lower limb fractures during a somewhat earlier Covid-19 wave from January 25 to March 25 compared with the preceding 4 years, which is in line with our findings (Wong and Cheung 2020). In London, there was a substantial decrease in the number of trauma referrals, admissions, and operations, suggesting that governmental recommendations actually had an effect on activity in the population (Park et al. 2020, Sugand et al. 2020).

We found no reduction in the number of ankle fractures among men, and this is difficult to explain. Maybe women adhered to the recommendations from the authorities to a greater extent. An increase in number of fractures was seen during the second 30-day period for the age group 30–49 years. A similar rebound but for hip fractures was shown in Finland by Ponkilainen et al. (2020).

A strength of our study is the fact that the time period of the Covid-19 pandemic in spring 2020 was compared with a mean for the same time period over the past 3 years. Most other studies have compared 2020 with 1 other year, which might lead to an overinterpretation of the differences seen for single years. Another strength is that it includes 7 different orthopedic departments in Sweden, while most other studies are single center (Haskel et al. 2020, Lubbe et al. 2020, Park et al. 2020).

A possible limitation of our study is that, even though the included departments have a history of rapid fracture registration entry in the SFR, the timeframe between the studied time period and data extraction from the SFR is short and, as a result, some fractures may not have been entered before data extraction.

In conclusion, we observed a decline in the number of ankle fractures during the 1st wave of the Covid-19 pandemic and the subsequent social distancing and reduction of activities in society. The reduction in the number of ankle fractures was greatest during the 1st 30-day period, among women and people aged > 70 years. This may indicate a greater adherence to government recommendations in these sub-groups and during the first period of the pandemic. Ankle fracture incidence may reflect the extent of lockdowns.
